# Public Knowledge and Awareness of Gallstone Disease and Its Association With Self-Reported Metabolic and Lifestyle Risk Factors Among Adults in Jeddah, Saudi Arabia

**DOI:** 10.7759/cureus.116851

**Published:** 2026-09-24

**Authors:** Sameh S Mohammed, Waleed Alrashidi, Ammar Ayash, Ammar Aldaadi, Fahad Baabdullah, Malik Alzahrani, Mansour Allhyani, Mohamed Seifalyazal

**Affiliations:** 1 Surgery, Ibn Sina National College for Medical Studies, Jeddah, SAU; 2 Surgery, Ain Shams University, Cairo, EGY; 3 Endocrine Surgery, Ibn Sina National College for Medical Studies, Jeddah, SAU; 4 Endocrine Surgery, Ain Shams University, Cairo, EGY

**Keywords:** adult, awareness, bariatric, gallstone, jeddah, knowledge, metabolic syndrome, obesity, risk factor, saudi

## Abstract

Background: Metabolic syndrome is a major, increasing global public health concern, including in Saudi Arabia, and is a well-established, significant modifiable risk factor for the development of cholesterol-based gallstone disease. Given the high prevalence of metabolic syndrome and gallstones in the Kingdom, assessing public knowledge and awareness of this specific link is crucial for effective prevention strategies.

Objective: This study aimed to assess public knowledge and awareness of gallstone disease among adults in Jeddah, Saudi Arabia, and to explore self-reported associations between gallstone history and selected metabolic and lifestyle risk factors, namely, diabetes mellitus, dyslipidemia, and physical activity. Metabolic syndrome itself was not objectively diagnosed in this study.

Methods: A cross-sectional survey method was utilised, distributing a self-administered, structured electronic questionnaire built on Google Forms (Google LLC, Mountain View, CA, USA) to a convenience sample of adults living in Jeddah. The questionnaire included sections on demographics, general awareness of gallstones, knowledge of risk factors, and participants' self-reported metabolic and lifestyle characteristics. Data analysis included descriptive statistics to evaluate the overall level of awareness and knowledge and bivariate inferential statistics to identify factors associated with higher knowledge scores and with self-reported gallstone history.

Results: Among 333 Jeddah residents analysed, 34 (10.2%) reported a prior diagnosis of gallstones on direct questioning; this rose to 37 (11.1%) when the small number of participants who were "not sure" of a formal diagnosis but reported having received specific gallstone treatment were included, as pre-specified. Of the 333 participants, 155 (46.5%) had a fair knowledge level, 73 (21.9%) had good knowledge, and 105 (31.5%) had a poor knowledge score (mean score 6.93±2.90 out of 12). A statistically significant association was observed between family history of gallstones and self-reported gallstone status (p<0.001), with 27 (73.0%) of gallstone-positive participants reporting a positive family history. Statistically significant associations were also observed between gallstone status and diabetes mellitus (p<0.0001), self-reported high cholesterol or triglycerides (p<0.001), and physical activity level (p=0.010). A statistically significant relation was observed between knowledge level and participants' age (p=0.0021), nationality (p=0.020), employment status (p=0.0042), marital status (p=0.0093), and body weight (p=0.040).

Conclusion: The adult population in Jeddah, Saudi Arabia, shows measurable knowledge gaps regarding gallstone disease, and self-reported gallstone history was associated with several self-reported metabolic and lifestyle factors, most notably family history, diabetes mellitus, and dyslipidemia. Targeted health education initiatives, particularly for the population subgroups identified here as having lower knowledge, may help address this gap; because this was a self-reported, cross-sectional convenience sample, these findings should be regarded as hypothesis-generating rather than as a basis for population-level prevalence estimates or causal claims.

## Introduction

Metabolic syndrome is a complex metabolic disorder that significantly increases the risk of atherosclerotic cardiovascular diseases affecting both central and peripheral vascular systems, including coronary artery disease, peripheral artery disease, myocardial infarction, and cerebrovascular events [1]. In addition, metabolic syndrome is strongly associated with an increased risk of type 2 diabetes mellitus and several malignancies [2]. According to the collaborative criteria established in 2009, the diagnosis of metabolic syndrome requires the presence of at least three of five components: increased waist circumference (>80 cm in women and >94 cm in men), elevated triglyceride levels (>150 mg/dL or receiving treatment for hypertriglyceridemia), reduced high-density lipoprotein cholesterol levels (<50 mg/dL in women and <40 mg/dL in men or receiving treatment), elevated blood pressure (≥130/85 mmHg or receiving antihypertensive therapy), and elevated fasting plasma glucose (≥100 mg/dL or receiving treatment for hyperglycemia) [3]. More recently, the 2022 updated criteria emphasised the central role of obesity by considering body mass index (BMI) >30 kg/m² or increased waist circumference (>88 cm in women and >102 cm in men) alongside two additional metabolic abnormalities as sufficient for diagnosis. This update highlights obesity as a cornerstone component in the development and progression of metabolic syndrome. It should be noted that these waist-circumference thresholds are not universally applicable across populations; population-specific cutoffs have been proposed for the Saudi population (from the SAUDI-DM study) that differ from the values above, reflecting variation in body-fat distribution by ethnicity, and this variation is relevant when interpreting metabolic syndrome criteria in a Saudi cohort such as the present one [4].

Sedentary lifestyle patterns and excessive caloric intake have contributed substantially to the increasing prevalence of obesity worldwide [2]. Obesity, defined as BMI >30 kg/m², affects approximately one-third of adults globally [5]. It is recognised as a chronic low-grade inflammatory state that contributes to metabolic disturbances, including dyslipidemia, insulin resistance, and hyperglycemia [6]. These metabolic alterations are also implicated in the development of gallstone disease, a common condition affecting approximately 10-20% of the global population, particularly among individuals with excessive body weight [7].

Central obesity is considered one of the major risk factors associated with gallstone formation [8,9], and the prevalence of gallstone disease is expected to increase with the continued rise in overweight and obesity rates worldwide [10]. Several mechanisms have been proposed to explain the association between obesity and gallstone formation. Insulin resistance, commonly observed in obese individuals, results in elevated plasma insulin levels [11], which stimulate the activity of 3-hydroxy-3-methylglutaryl coenzyme A (HMG-CoA) reductase, leading to increased hepatic cholesterol synthesis and cholesterol hypersecretion into bile [12,13]. Since more than 90% of gallstones are cholesterol-based, disturbances in cholesterol metabolism play a crucial role in their development [14]. Furthermore, alterations in bile lipid transport, cholesterol esterification pathways, regulatory signalling mechanisms, and gallbladder motility may contribute to cholesterol gallstone formation in both obese and non-obese individuals [15]. However, additional complex mechanisms are likely involved in the pathophysiology of gallstone disease [16-18].

Although surgical intervention remains the definitive treatment for symptomatic gallstone disease with relatively low mortality rates, controlling modifiable risk factors, including obesity, unhealthy dietary patterns, physical inactivity, and medication-related factors, remains essential to reduce disease burden and healthcare costs [19].

In the Kingdom of Saudi Arabia (KSA), the prevalence of gallstone disease reflects the global trend. A study conducted in 2017 reported a prevalence of 8.6% among the Saudi population, with a strong association between gallstone disease and metabolic syndrome-related risk factors, particularly obesity and physical inactivity. Among individuals with gallstone disease, 67.4% were reported to be obese [20]. Despite some level of public awareness regarding gallstone disease and its general risk factors, specific knowledge regarding the relationship between gallstone formation and metabolic syndrome remains insufficient among the Saudi population [21]. Improving awareness of these interconnected risk factors may contribute to preventive strategies, particularly in populations with high obesity prevalence such as Saudi Arabia.

Therefore, this study aimed to assess public knowledge and awareness of gallstone disease and to explore self-reported associations between gallstone history and selected metabolic and lifestyle risk factors among adults in Jeddah, Saudi Arabia. This study assesses perceived and self-reported associations rather than the underlying pathophysiological mechanisms linking metabolic syndrome and gallstone formation, which were outside its scope.

## Materials and methods

Setting and design

A cross-sectional study was conducted among adults residing in Jeddah, Saudi Arabia, from 12 October to 22 November 2025. The study aimed to assess public knowledge and awareness of gallstone disease and to explore self-reported associations between gallstone history and selected metabolic and lifestyle risk factors among the adult population. Metabolic syndrome itself was not objectively assessed in this study, as waist circumference, blood pressure, fasting plasma glucose, and lipid profile were not directly measured; instead, self-reported diabetes mellitus, self-reported high cholesterol or triglycerides, self-reported physical activity, and body mass index derived from self-reported height and weight were used as proxy metabolic and lifestyle variables.

Study population

Participants were eligible if they were aged 18 years or older, currently residing in Jeddah, Saudi Arabia, and able to complete an online questionnaire in either Arabic or English. Participants who did not currently reside in Jeddah, or who submitted incomplete questionnaire responses, were excluded from the analysis.

Sampling method and sample size

A convenience non-probability sampling technique was used for participant recruitment. The required sample size was calculated based on a previous study reporting an estimated prevalence of gallstone disease in Saudi Arabia of 11.7% [22]. Considering a 95% confidence level, a 5% margin of error, and a 15% anticipated non-response rate, the minimum required sample size was estimated at 188 participants.

Study instrument

Data were collected using a structured questionnaire adapted from previously published studies [23,24]. The questionnaire was reviewed and modified to align with the objectives of the current study. No pilot study was conducted before data collection. The original questionnaire sources and authors were acknowledged according to the referenced studies [23,24].

The questionnaire was self-administered and distributed electronically using Google Forms (Google LLC, Mountain View, CA, USA), a free, cloud-based online survey and data-collection platform (accessed at forms.google.com). The electronic questionnaire link was disseminated through multiple social media platforms between 12 October and 22 November 2025. Responses were automatically compiled by Google Forms into a linked spreadsheet, which was subsequently exported to Microsoft Excel (Microsoft Corporation, Redmond, WA, USA) for data cleaning and statistical analysis. It consisted of four sections.

The first section collected participants' sociodemographic and academic characteristics, including age, sex, nationality, educational level, employment status, and marital status. Anthropometric information, including self-reported weight and height, was also collected to calculate BMI.

The second section assessed participants' medical history, including previous diagnosis of gallstone disease, received treatment, family history of gallstones, history of diabetes mellitus, history of dyslipidemia, and physical activity patterns.

The third section evaluated participants' previous awareness of gallstone disease and assessed their general knowledge regarding gallstone-related information, including risk factors, clinical manifestations, diagnostic methods, preventive measures, treatment options, and possible complications.

The fourth section assessed participants' perception of specific risk factors associated with gallstone disease, including lifestyle- and metabolic-related factors. The complete, final questionnaire administered to participants, reconciled with the items analysed in the Results section, is provided in the Appendices.

Gallstone disease status, family history of gallstones, diabetes mellitus, dyslipidemia, physical activity, and anthropometric data (height and weight) were all based entirely on participant self-report; none were verified against medical records, laboratory testing, imaging, or direct physical measurement. Accordingly, throughout this manuscript, "gallstone prevalence" and "gallstone status" refer strictly to self-reported previous diagnosis of gallstones (or, where stated, self-reported diagnosis together with a reported history of specific treatment), not to a clinically or radiologically confirmed diagnosis, and all other self-reported variables should be interpreted with the same caveat.

Statistical analysis

Knowledge of gallstone disease was assessed using the 12 knowledge items listed in Figure 1. Each item was scored 1 point for the pre-specified correct response shown below and 0 points for any incorrect response or "I don't know", yielding a summary knowledge score ranging from 0 to 12.

**Figure 1:**
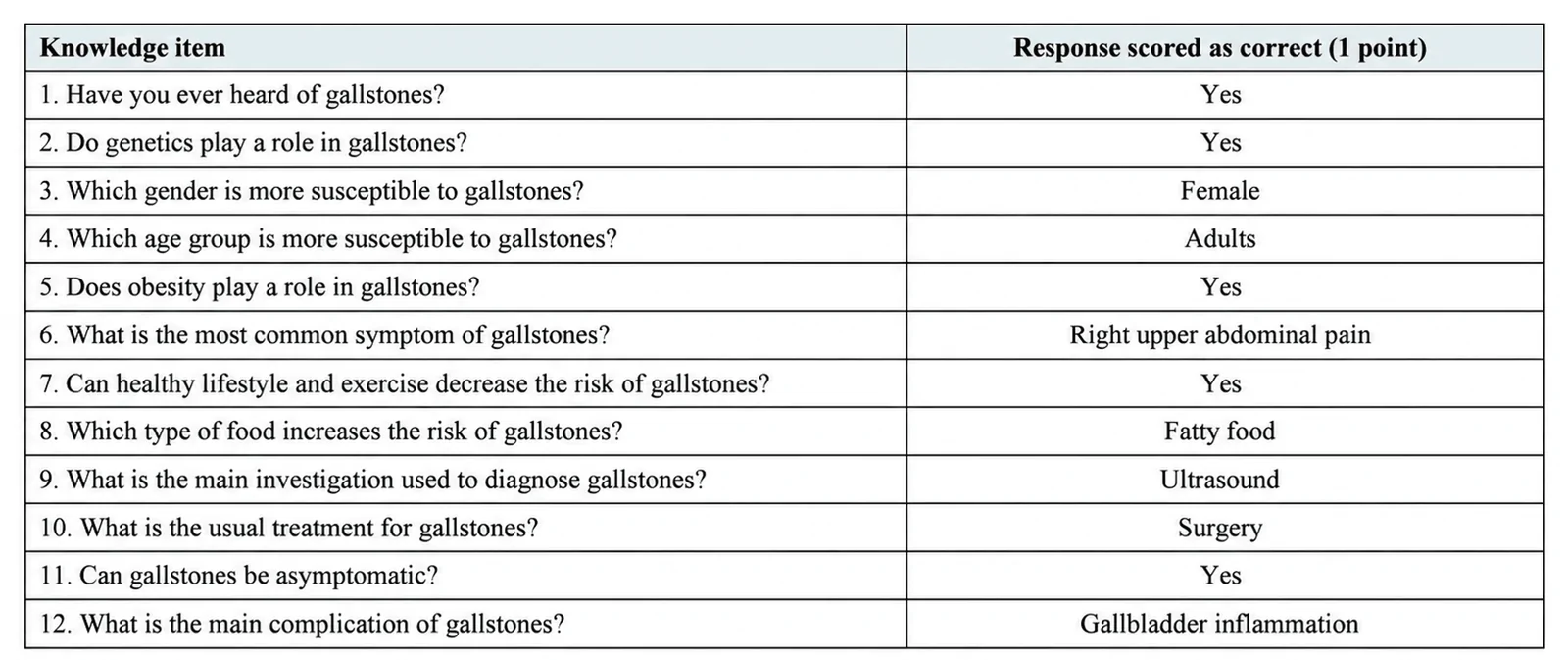
12 knowledge items used in assessing knowledge of gallstone disease Knowledge-item scoring key (1 point per item for the response shown; 0 points for any other response, including "I don't know"; maximum total score=12).

Based on this 0-12 score, participants were categorised as having poor (score 0-5, i.e., <50% correct), fair (score 6-9, 50-75% correct), or good (score 10-12, >75% correct) knowledge; these percentage-based cutoffs are a conventional, pre-specified categorisation of overall test performance and were applied consistently to all participants. Gallstone status for the bivariate analyses was defined as a literal "Yes" response to "Have you ever been diagnosed with gallstones?", together with the small number of participants who answered "Not sure" to that question but who separately reported having received a specific gallstone treatment (surgery, medication, or watchful waiting), who were classified as gallstone-positive on that basis; this reclassification rule and the resulting counts are stated explicitly in the Results.

Data were collected, coded, and analysed using IBM SPSS Statistics for Windows, V. 27.0 (released 2020, IBM Corp., Armonk, NY, USA). Categorical variables were presented as frequencies and percentages. The chi-squared test was used to assess associations between categorical variables. When more than 20% of cells had an expected count of <5, Monte Carlo correction (based on 20,000 permutations of the observed table or Fisher's exact test for 2×2 tables) was applied. Continuous variables were tested for normality using the Kolmogorov-Smirnov test. Normally distributed quantitative variables were presented as mean and standard deviation, while non-normally distributed variables were expressed as median and interquartile range (IQR), along with minimum and maximum values. The Kruskal-Wallis test was used to compare non-normally distributed quantitative variables among more than two groups. Statistical significance was determined at a p-value of <0.05. Given the small number of confirmed gallstone-positive cases after data verification (see Results), multivariable-adjusted regression analysis was not performed; this is discussed further in the Limitations section below.

Ethical considerations

Ethical approval was obtained from the Ibn Sina National College (ISNC) Institutional Research Review Board prior to the start of participant recruitment and data collection (approval number: IRRB-02-10022025; protocol identification number: 030MP/CR09022025; date of approval: 10 February 2025). Data collection took place between 12 October and 22 November 2025, entirely after ethical approval had been granted. Written informed consent was obtained electronically from all participants before completing the questionnaire. The first page of the survey included an explanation of the study objectives and a consent statement, and only participants who agreed to participate proceeded to complete the questionnaire.

Participation was voluntary, and participants were informed that they could withdraw from the study at any time without consequences. All collected data were used solely for scientific research purposes. Participant anonymity and confidentiality were maintained throughout all stages of the study.

## Results

Participants' current residency distribution

Among 389 respondents who accessed the questionnaire, 333 (85.6%) participants were currently residing in Jeddah and were eligible to complete the survey, while 56 (14.4%) participants were excluded because they resided outside Jeddah, in line with the study's eligibility criteria (Table 1).

**Table 1:** Distribution of the studied cases according to their current residency (n = 389)

Do you currently live in Jeddah?	No. (%)
Yes	333 (85.6%)
No	56 (14.4%)

Participants' demographic characteristics and physical activity

Among the 333 valid respondents, 145 (43.5%) were young adults aged 18-25 years, and 129 (38.7%) were aged 26-35 years. The remaining participants were distributed among those aged 36-45 (47, 14.1%) and those older than 45 years (12, 3.6%). The study population included 172 (51.7%) males and 161 (48.3%) females. Most participants were Saudi nationals (304, 91.3%), and regarding marital status, the majority of participants were single (239, 71.8%), while 91 (27.3%) were married. Participants were generally well educated, with 262 (78.7%) having a university education. Among all participants, 153 (45.9%) were employed.

Participants' anthropometric characteristics showed a mean weight of 71.63±20.71 kg and a mean height of 166.4±10.56 cm. These self-reported measurements were used to calculate BMI, with a mean BMI of 25.43±5.73 kg/m² (Table 2).

**Table 2:** Distribution of participants regarding their sociodemographic characteristics and anthropometric data (N=333)

Participant information	No. (%)
Age (years)	
18-25	145 (43.5%)
26-35	129 (38.7%)
36-45	47 (14.1%)
45+	12 (3.6%)
Gender	
Male	172 (51.7%)
Female	161 (48.3%)
Nationality	
Saudi	304 (91.3%)
Non-Saudi	29 (8.7%)
Educational background	
High school	33 (9.9%)
University	262 (78.7%)
Postgraduate	26 (7.8%)
Other	12 (3.6%)
Employment	
Employed	153 (45.9%)
Student	128 (38.4%)
Unemployed	27 (8.1%)
Homemaker	21 (6.3%)
Retired	4 (1.2%)
Marital status	
Single	239 (71.8%)
Married	91 (27.3%)
Other	3 (0.9%)
Height (cm)	
Min.-max.	138.0-192.0
Mean±SD	166.4±10.56
Median (IQR)	167.0 (158.0-175.0)
Weight (kg)	
Min.-max.	28.0-145.0
Mean±SD	71.63±20.71
Median (IQR)	68.0 (56.0-84.0)

Participants' medical background

The self-reported prevalence of a prior gallstone diagnosis among study participants was 10.2% (34/333). Among the 34 participants who reported a prior gallstone diagnosis, 26 (76.5%) had undergone surgical intervention, four (11.8%) received medications, and four (11.8%) were on watchful waiting. A positive family history of gallstones was reported by 129 (38.7%) participants. Most participants reported no history of diabetes mellitus (299, 89.8%), and 224 (67.3%) reported no history of dyslipidemia. Regarding physical activity patterns, 177 (53.2%) participants reported exercising 1-2 days per week, 131 (39.3%) exercised 3-5 days per week, and 25 (7.5%) exercised 6-7 days per week (Table 3).

**Table 3:** Distribution of participants regarding their self-reported medical background (N=333) (*) In the version of this manuscript previously submitted for peer review, item 1 of this table was reported as 154 Yes (46.2%)/177 No (53.2%)/2 Not sure (0.6%), with a treatment breakdown of 30/11/22/91. Re-verification against the source dataset, undertaken in response to the reviewers' concern regarding the plausibility of this figure, identified that the original tabulation had inadvertently counted responses to the adjacent "If Yes, treatment received" follow-up field (154 non-blank entries in total) rather than direct responses to the diagnosis question itself; a substantial proportion of those entries came from participants who had actually answered "No" to the diagnosis question, most likely reflecting a survey skip-logic or data-export artefact rather than an error in the participants' own responses. This has been corrected throughout the manuscript to reflect participants' actual responses to "Have you ever been diagnosed with gallstones?", 34 Yes (10.2%), 294 No (88.3%), 4 Not sure (1.2%), and 1 not stated (0.3%), with the treatment breakdown restricted to the 34 confirmed Yes respondents. This correction is also reflected in Tables 6-8 and in the Abstract, Results, and Discussion.

Q	Medical background (self-report)	No. (%)
1	Have you ever been diagnosed with gallstones?	
	Yes	34 (10.2%)*
	No	294 (88.3%)
	Not sure	4 (1.2%)
	Not stated	1 (0.3%)
2	If yes, treatment received (n=34)	
	Surgery	26 (76.5%)
	Medications	4 (11.8%)
	Watchful waiting	4 (11.8%)
	Other	0 (0.0%)
3	Do you have a close family member with gallstones?	
	Yes	129 (38.7%)
	No	166 (49.8%)
	I don't know	38 (11.4%)
4	Do you have diabetes mellitus?	
	Yes	28 (8.4%)
	No	299 (89.8%)
	Not sure	6 (1.8%)
5	Have you ever been told you have high cholesterol or triglycerides?	
	Yes	95 (28.5%)
	No	224 (67.3%)
	Not sure	14 (4.2%)
6	Physical activity in a typical week?	
	1-2 days	177 (53.2%)
	3-5 days	131 (39.3%)
	6-7 days	25 (7.5%)

Participants' knowledge and awareness about gallstones

The current study demonstrated that 301 (90.4%) respondents had previously heard about gallstones. Participants showed awareness regarding several general aspects of gallstone disease, including genetic predisposition, gender susceptibility, the relationship between obesity and gallstone formation, common symptoms, preventive lifestyle measures, dietary risk factors, diagnostic methods, treatment options, and complications.

Specifically, 125 (37.5%) participants recognised the role of genetics in gallstone development, 96 (28.8%) identified females as having higher susceptibility, and 151 (45.3%) identified adults as the age group more commonly affected. Additionally, 211 (63.4%) participants identified obesity as a risk factor, while 210 (63.1%) recognised right upper abdominal pain as the most common symptom. A total of 275 (82.6%) participants agreed that healthy lifestyle practices and exercise may reduce the risk of gallstones, and 280 (84.1%) identified consumption of fatty foods as a contributing factor. Regarding diagnosis and treatment, 131 (39.3%) recognised ultrasound as the main diagnostic investigation, and 217 (65.2%) identified surgery as the usual treatment option. Furthermore, 129 (38.7%) participants recognised that gallstones can be asymptomatic, while 182 (54.7%) identified gallbladder inflammation as a major complication (Table 4).

**Table 4:** Distribution of the studied cases according to knowledge and awareness about gallstones (N=333)

Q	Knowledge and awareness about gallstones	No. (%)
1	Have you ever heard of gallstones?	
	Yes	301 (90.4%)
	No	32 (9.6%)
2	Do genetics play a role in gallstones?	
	Yes	125 (37.5%)
	No	65 (19.5%)
	I don't know	143 (42.9%)
3	Which gender is more susceptible to gallstones?	
	Male	56 (16.8%)
	Female	96 (28.8%)
	Both equally	76 (22.8%)
	I don't know	105 (31.5%)
4	Which age group is more susceptible to gallstones?	
	Adults	151 (45.3%)
	Elderly	108 (32.4%)
	Children	3 (0.9%)
	I don't know	71 (21.3%)
5	Does obesity play a role in gallstones?	
	Yes	211 (63.4%)
	No	24 (7.2%)
	I don't know	98 (29.4%)
6	What is the most common symptom of gallstones?	
	Right upper abdominal pain	210 (63.1%)
	Nausea/vomiting	35 (10.5%)
	Yellowish skin or eyes	20 (6.0%)
	I don't know	68 (20.4%)
7	Can healthy lifestyle and exercise decrease the risk of gallstones?	
	Yes	275 (82.6%)
	No	8 (2.4%)
	I don't know	50 (15.0%)
8	Which type of food increases the risk of gallstones?	
	Fatty food	280 (84.1%)
	Vegetables	8 (2.4%)
	I don't know	45 (13.5%)
9	What is the main investigation used to diagnose gallstones?	
	Ultrasound	131 (39.3%)
	X-ray	28 (8.4%)
	CT scan	53 (15.9%)
	I don't know	121 (36.3%)
10	What is the usual treatment for gallstones?	
	Surgery	217 (65.2%)
	Medications	53 (15.9%)
	Herbal remedies	7 (2.1%)
	I don't know	56 (16.8%)
11	Can gallstones be asymptomatic?	
	Yes	129 (38.7%)
	No	86 (25.8%)
	I don't know	118 (35.4%)
12	What is the main complication of gallstones?	
	Gallbladder inflammation	182 (54.7%)
	Kidney stones	40 (12.0%)
	Pancreatitis	16 (4.8%)
	I don't know	95 (28.5%)

Participants' perception regarding risk factors of gallstones

Most participants recognised several gallstone risk factors. A total of 129 (38.7%) participants reported a family history of gallstones in a direct relative, while 181 (54.4%) considered age a risk factor for gallstones, and 265 (79.6%) believed obesity increases the risk of gallstone formation. Dyslipidemia-related factors were also widely recognised, with 251 (75.4%) participants identifying high cholesterol levels and 249 (74.8%) identifying high triglyceride levels as risk factors. In addition, 153 (45.9%) participants considered diabetes mellitus to be associated with gallstone development, while 146 (43.8%) recognised low high-density lipoprotein levels as a potential risk factor (Table 5).

**Table 5:** Distribution of the studied cases according to perception about risk factors of gallstones (N=333) (*) In the original submission, item 1 of this table was reported as 130 (39.0%)/170/33; this has been corrected to 129 (38.7%)/170 (51.1%)/34 (10.2%) after re-verification against the source dataset, aligning it with the identical family history question reported in Table 3 (129, 38.7%). HDL: high-density lipoprotein

Q	Risk factor perception	Yes	No	I don't know
1	Do you have a family history of gallstones (in direct relatives)?	129 (38.7%)*	170 (51.1%)	34 (10.2%)
2	Do you think age is a risk factor for gallstones?	181 (54.4%)	86 (25.8%)	66 (19.8%)
3	Do you think obesity increases the risk of gallstones?	265 (79.6%)	19 (5.7%)	49 (14.7%)
4	Do you think high cholesterol is a risk factor for gallstones?	251 (75.4%)	17 (5.1%)	65 (19.5%)
5	Do you think high triglycerides increase the risk of gallstones?	249 (74.8%)	21 (6.3%)	63 (18.9%)
6	Do you think diabetes mellitus is a risk factor for gallstones?	153 (45.9%)	69 (20.7%)	111 (33.3%)
7	Do you think low HDL ("good cholesterol") is a risk factor for gallstones?	146 (43.8%)	42 (12.6%)	145 (43.5%)

Knowledge score regarding gallstones

The assessment of participants' overall knowledge level regarding gallstones revealed that 155 (46.5%) participants had a fair knowledge level, 73 (21.9%) had good knowledge, and 105 (31.5%) had poor knowledge scores. The mean knowledge score was 6.93±2.90 out of 12, with a median score of 7.0 (IQR: 5.0-9.0) (Table 6).

**Table 6:** Distribution of the studied cases according to score of knowledge and awareness about gallstones (N=333) In the version of this manuscript previously submitted for peer review, this table reported a mean knowledge score of 7.26±3.15 and a poor/fair/good split of 88 (26.4%)/155 (46.5%)/90 (27.0%). Re-verification confirmed that every one of the 12 individual knowledge-item counts in Table 4 is correct and reproduces exactly from the source dataset but that the previously published aggregate score was arithmetically inconsistent with those same item-level counts (the maximum mean achievable by summing the 12 confirmed item-level correct-response counts is 6.93, not 7.26). This has been corrected using the scoring key defined in the Statistical Analysis subsection: mean score 6.93±2.90 (median 7.0; IQR 5.0-9.0), with 105 (31.5%) poor, 155 (46.5%) fair, and 73 (21.9%) good; the fair category count (155) is unchanged. This correction is also reflected in Table 8 and in the Abstract, Results, and Discussion.

	No. (%)
Level	
Poor (<50%)	105 (31.5%)
Fair (50-75%)	155 (46.5%)
Good (>75%)	73 (21.9%)
Total score (0-12)	
Min.-max.	0.0-12.0
Mean±SD	6.93±2.90
Median (IQR)	7.0 (5.0-9.0)
Average score (0-1)	
Min.-max.	0.0-1.0
Mean±SD	0.58±0.24
Median (IQR)	0.58 (0.42-0.75)
% score	
Min.-max.	0.0-100.0
Mean±SD	57.75±24.17
Median (IQR)	58.33 (41.67-75.00)

Relationship between gallstone prevalence and participants' medical background

For this bivariate analysis, gallstone-positive status was defined as a literal "Yes" response to "Have you ever been diagnosed with gallstones?" (Table 3), together with the three participants who initially answered "Not sure" but who subsequently reported having received treatment (surgery, medication, or watchful waiting) for gallstones, who were classified within the gallstone-positive group, consistent with their reported treatment history. This yielded an analytic denominator of 37 gallstone-positive and 295 gallstone-negative participants (n=332, excluding one participant who did not answer this question) for the associations presented below.

We observed a statistically significant association between family history of gallstones and gallstone status (p<0.001), with 27 (73.0%) gallstone-positive participants reporting a positive family history, compared with 102 (34.6%) gallstone-negative participants. Furthermore, a statistically significant association was detected between diabetes mellitus and gallstone status (p<0.0001); 16 (43.2%) of gallstone-positive participants reported diabetes mellitus, compared with only 12 (4.1%) of gallstone-negative participants. Similarly, a significant association was observed between gallstone status and self-reported high cholesterol or triglyceride levels (p<0.001), reported by 25 (67.6%) of gallstone-positive versus 70 (23.7%) of gallstone-negative participants. Physical activity level was also significantly associated with gallstone status (p=0.010): gallstone-positive participants were more concentrated in the lowest activity category (27, 73.0%, exercising 1-2 days/week) than gallstone-negative participants (150, 50.8%); given the cross-sectional design, this association should be described as gallstone status being associated with lower physical activity level, not as physical activity causing or preventing gallstone formation, and the pattern across activity categories was not strictly monotonic. Notably, despite the much smaller, corrected size of the gallstone-positive group compared with the previously reported figure, all four of these bivariate associations remained statistically significant (Table 7).

**Table 7:** Relation between gallstone status and medical background (n=332) In the version of this manuscript previously submitted for peer review, this table used an analytic denominator of 156 gallstone-positive/177 gallstone-negative participants, derived from the erroneous Table 3 prevalence figure described in the Table 3 note above. Using the corrected gallstone-positive group (n=37), all four associations were re-tested; each remains statistically significant, although the underlying counts, chi-squared statistics, and (for diabetes) the correction method used (Monte Carlo simulation, as more than 20% of cells had an expected count of <5) have changed as shown above.

Medical background (self-report)	Yes (n=37)	No (n=295)	χ²	P
Do you have a close family member with gallstones?				
Yes	27 (73.0%)	102 (34.6%)	20.86*	<0.001*
No	7 (18.9%)	159 (53.9%)		
I don't know	3 (8.1%)	34 (11.5%)		
Do you have diabetes mellitus?				
Yes	16 (43.2%)	12 (4.1%)	65.93*	MC p<0.0001*
No	20 (54.1%)	278 (94.2%)		
Not sure	1 (2.7%)	5 (1.7%)		
Have you ever been told you have high cholesterol or triglycerides?				
Yes	25 (67.6%)	70 (23.7%)	31.00*	<0.001*
No	11 (29.7%)	212 (71.9%)		
Not sure	1 (2.7%)	13 (4.4%)		
Physical activity in a typical week?				
1-2 days	27 (73.0%)	150 (50.8%)	9.21*	0.010*
3-5 days	6 (16.2%)	124 (42.0%)		
6-7 days	4 (10.8%)	21 (7.1%)		

Relationship between knowledge level and demographic characteristics

A statistically significant association was observed between participants' knowledge level and age (p=0.0021), with 51 (48.6%) participants aged 26-35 years classified as having poor knowledge regarding gallstones. Significant associations were also detected between knowledge level and nationality (p=0.020), employment status (p=0.0036), marital status (p=0.0093), and body weight (p=0.028), with better knowledge associated with student status and with lower body weight. No statistically significant associations were identified between knowledge level and gender (p=0.098), educational background (p=0.271), or height (p=0.353) (Table 8).

**Table 8:** Relation between level of knowledge and demographic data (n=332-333; see note *) (*) This table uses the corrected knowledge-level categorisation from Table 6 (poor 105/fair 155/good 73). Employment and marital status are reported at n=332 (one participant did not answer these items); all other rows are reported at n=333. Height (n=318) and weight (n=320) exclude a small number of self-reported values that could not be reliably parsed as physiologically plausible measurements (see Statistical analysis). H: Kruskal-Wallis test statistic; MC: Monte Carlo-simulated p-value

Demographic data	Poor (n=105)	Fair (n=155)	Good (n=73)	χ²/p
Age (years)				
18-25	35 (33.3%)	72 (46.5%)	38 (52.1%)	χ²=20.703*/0.0021*
26-35	51 (48.6%)	48 (31.0%)	30 (41.1%)	
36-45	18 (17.1%)	25 (16.1%)	4 (5.5%)	
45+	1 (1.0%)	10 (6.5%)	1 (1.4%)	
Gender				
Male	63 (60.0%)	72 (46.5%)	37 (50.7%)	χ²=4.636/0.098
Female	42 (40.0%)	83 (53.5%)	36 (49.3%)	
Nationality				
Saudi	91 (86.7%)	141 (91.0%)	72 (98.6%)	χ²=7.790/0.020*
Non-Saudi	14 (13.3%)	14 (9.0%)	1 (1.4%)	
Educational background				
High school	13 (12.4%)	13 (8.4%)	7 (9.6%)	χ²=7.577/0.271
University	80 (76.2%)	125 (80.6%)	57 (78.1%)	
Postgraduate	8 (7.6%)	9 (5.8%)	9 (12.3%)	
Other	4 (3.8%)	8 (5.2%)	0 (0.0%)	
Employment				
Employed	58 (55.2%)	68 (43.9%)	26 (36.1%)	χ²=21.971*/MC p=0.0036*
Student	27 (25.7%)	60 (38.7%)	41 (56.9%)	
Unemployed	13 (12.4%)	12 (7.7%)	2 (2.8%)	
Homemaker	6 (5.7%)	13 (8.4%)	2 (2.8%)	
Retired	1 (1.0%)	2 (1.3%)	1 (1.4%)	
Marital status				
Single	67 (63.8%)	112 (72.3%)	60 (83.3%)	χ²=13.17*/MC p=0.0093*
Married	35 (33.3%)	43 (27.7%)	12 (16.7%)	
Other	3 (2.9%)	0 (0.0%)	0 (0.0%)	
Height (cm), mean±SD	168.05±10.52	166.44±10.14	165.38±10.47	H=2.083/0.353
Weight (kg), mean±SD	77.04±25.03	72.55±20.74	67.65±21.90	H=7.131/0.028*

## Discussion

Gallstone disease is a common gastrointestinal disorder associated with substantial healthcare burden, including increased hospital admissions, healthcare costs, and morbidity rates [25]. The prevalence of gallstones varies worldwide, with rates reaching up to 20% in some European and US populations, and a recent global systematic review and meta-analysis spanning studies from 2000 to 2024 similarly reported wide regional variation, generally in the range of 5-25% depending on population and diagnostic method [13,26]. This variation may be attributed to differences in genetic susceptibility, environmental factors, lifestyle patterns, and metabolic conditions [26]. Given the increasing prevalence of obesity and metabolic syndrome-related factors, understanding public knowledge regarding gallstone disease and its modifiable risk factors is essential for developing effective preventive strategies.

The current study assessed the level of knowledge and awareness regarding gallstone disease and its associated risk factors among 333 adults in Jeddah, Saudi Arabia. The findings demonstrated that only 21.9% of participants achieved a good knowledge score, whereas 46.5% had a fair knowledge level and 31.5% had poor knowledge regarding gallstones. These findings are broadly consistent with those reported by Almetrek et al. [21], who found that only 5.6% of participants had high knowledge and 32.6% had poor knowledge scores; we verified these two figures directly against the original source and confirmed them as reported (this citation was numbered [25] in the version of this manuscript previously submitted for peer review, which was an in-text citation-numbering error, now corrected to [21]; Almetrek et al.'s findings are distinct from, and should not be confused with, the family history findings of Mirghani et al. [27] discussed separately below). Similarly, a previous study conducted in a different region reported limited public knowledge regarding gallbladder diseases and their associated risk factors [28]. However, another Saudi Arabian study involving 429 participants reported higher awareness levels, with 65.3% of participants demonstrating knowledge regarding different aspects of gallstone disease, 73% having heard about gallstones and related complications, and 72.8% being familiar with the definition of gallstones [29]. The variation between studies may be related to differences in study populations, educational backgrounds, and methods used to assess knowledge.

In the present study, the self-reported prevalence of a prior gallstone diagnosis among participants was 10.2% (34/333), rising to 11.1% (37/332) when participants who were "not sure" of a formal diagnosis but reported specific gallstone treatment were included, as pre-specified; this explicitly refers to self-reported, previously diagnosed gallstone disease, not a clinically or radiologically confirmed population prevalence. This figure is broadly comparable to, if somewhat higher than, the 8.6% prevalence reported by Alishi et al. [20] among the general population in Riyadh, Saudi Arabia, in 2017 and sits within the range reported by recent global meta-analyses [13]. As with any convenience-sampled online survey, self-selection may still have influenced this figure in either direction, for example, individuals with a personal or family history of gallstones may have been more likely to complete a gallstone-themed survey, and, because this prevalence was self-reported and not independently verified against imaging or medical records, it should be interpreted as an estimate of self-reported diagnosis in this convenience sample rather than as a population-representative clinical prevalence. Furthermore, 38.7% of participants reported a family history of gallstones. Most participants reported no history of diabetes mellitus or dyslipidemia and reported predominantly low-frequency physical activity. A comparably high proportion of participants reported a family history of gallstones in the Saudi literature: Mirghani et al. [27] similarly reported that 39.9% of participants had a positive history of gallstones, either directly or in a family member.

Regarding awareness of gallstone risk factors, participants demonstrated relatively good recognition of several important contributing factors. Most participants identified obesity as a risk factor, and many recognised the role of unhealthy dietary habits, dyslipidemia, elevated cholesterol levels, elevated triglyceride levels, and diabetes mellitus in gallstone development. These findings indicate a reasonable level of awareness regarding the metabolic and lifestyle-related factors associated with gallstones. However, awareness of certain factors, such as genetic predisposition and low high-density lipoprotein levels, remained limited.

Previous Saudi studies have reported variable levels of awareness regarding gallstone risk factors. Mirghani et al. [27] demonstrated that genetic factors were recognised by only 12.7% of participants, while female sex was identified as a substantial risk factor by a substantial proportion of respondents. In the same study, high triglyceride levels, high cholesterol levels, and obesity were reported as important perceived causes of gallstone formation by 48.6%, 46.1%, and 38.4% of participants, respectively. Another study found that 59% of participants believed gallstones predominantly affected females, while only 13.4% recognised genetic factors as contributing causes [28]. Furthermore, approximately 65% of participants in another investigation acknowledged hereditary factors as influencing gallstone formation, and 68% recognised that females have a higher risk compared with males [29].

The current study identified statistically significant bivariate associations between self-reported gallstone status and several self-reported metabolic and familial factors. A significant association was observed between a positive family history and gallstone status, consistent with a possible contribution of genetic or familial factors, although this cross-sectional design cannot establish the direction or causal nature of this relationship. Additionally, significant associations were observed between gallstone status and diabetes mellitus, dyslipidemia, and physical activity level. These associations are consistent with the broader metabolic risk factor literature on gallstone disease and highlight lifestyle modification as a plausible focus for preventive efforts, but as bivariate, unadjusted findings in a self-reported cross-sectional sample, they should be read as associative rather than as evidence for a specific causal or pathogenic mechanism.

A significant association was also observed between participants' knowledge level and age, with 48.6% of participants aged 26-35 years demonstrating poor knowledge regarding gallstones. This age-related knowledge gap is a descriptive, associative finding regarding public awareness in this sample; it is conceptually distinct from work such as Pęczuła et al. [30], a genetics review addressing which inherited factors predispose to gallstone formation, rather than public knowledge or awareness of gallstone disease, and the two should not be read as convergent or "similar" findings.

Overall, the findings of this study highlight the presence of knowledge gaps regarding gallstone disease and self-reported associations with several metabolic and lifestyle factors among adults in Jeddah, Saudi Arabia. Improving public awareness through targeted health education programs focusing on obesity prevention, healthy dietary habits, physical activity, and metabolic risk awareness may help address this gap; because the underlying associations are drawn from a self-reported, cross-sectional convenience sample, this recommendation should be regarded as a reasonable, hypothesis-generating direction for public health efforts rather than as a claim of demonstrated causal benefit.

Limitations

This study has several limitations that should be considered when interpreting the findings. First, the cross-sectional design precludes any causal inference between the self-reported metabolic and lifestyle factors examined and gallstone disease; only statistical associations could be demonstrated, and language throughout this manuscript has been revised to reflect this consistently. Second, a convenience, non-probability sampling method was used, and the questionnaire was distributed exclusively online through social media platforms; this approach may have introduced selection bias toward younger, more educated, and more digitally engaged individuals, as reflected in the predominantly young (82.2% aged 18-35 years) and highly educated (86.5% university- or postgraduate-educated) study sample, which limits the generalisability of the findings to the broader adult population of Jeddah. This same self-selection process may also have influenced who chose to complete a gallstone-themed survey in the first place, for example, individuals with a personal or family history of gallstones may have been more likely to participate, and so may have affected the self-reported prevalence estimate itself, not only the generalisability of the knowledge findings; this is a further reason the prevalence reported here should be read as specific to this self-selected sample rather than as a population estimate. Third, gallstone diagnosis, family history, and comorbidities (diabetes mellitus and dyslipidemia) were based entirely on self-report without verification against medical records or imaging studies, introducing the potential for recall bias, social-desirability bias, and misclassification; "gallstone prevalence" throughout this manuscript should accordingly be read explicitly as unverified self-report, not a clinically confirmed diagnosis. Fourth, anthropometric data (height and weight) were self-reported rather than directly measured, which may have affected the accuracy of the derived BMI. Fifth, although the questionnaire was adapted from previously published, related instruments, it was not formally piloted or psychometrically validated (e.g., for internal consistency or test-retest reliability) in this population before use. Sixth, multivariable-adjusted analysis of gallstone status and knowledge level was not performed; once gallstone status was corrected to reflect the true number of self-reported/treated cases in the sample (n=37; 11.1%), this outcome was too rare relative to the number of candidate covariates to support a stable multivariable model, so we restricted our analysis to bivariate comparisons, which nonetheless identified several statistically robust associations; future studies with larger samples, particularly ones powered around the true, lower prevalence of self-reported gallstone disease observed here, are needed to determine which of these associations remain independent after adjustment for confounding. Finally, the study was restricted to adults residing in a single city (Jeddah), which may limit the generalisability of the findings to other regions of Saudi Arabia with different demographic, cultural, or healthcare-access profiles. Future studies employing random or stratified sampling, clinically or radiologically confirmed gallstone diagnosis, validated knowledge instruments, multivariable-adjusted analysis, and multi-centre designs are recommended to validate and extend these findings.

## Conclusions

The findings of this study demonstrated a considerable gap between general awareness of gallstone disease and specific knowledge regarding its self-reported associations with metabolic and lifestyle risk factors; metabolic syndrome itself was not objectively assessed. Although most participants had previously heard about gallstones, a substantial proportion showed inadequate knowledge regarding important risk factors, preventive strategies, clinical manifestations, and treatment options. Furthermore, limited understanding was observed regarding the perceived relationship between obesity, metabolic factors, and gallstone development.

These findings highlight the need for targeted public health initiatives aimed at improving awareness of gallstone disease and its self-reported metabolic and lifestyle risk factors. Educational campaigns should emphasise the role of obesity, unhealthy dietary patterns, physical inactivity, and related metabolic factors in gallstone risk. Promoting healthy lifestyle behaviours and improving public understanding of preventive measures may help reduce this knowledge gap; given the self-reported, cross-sectional, convenience-sampled design of this study, these recommendations should be regarded as reasonable directions for targeted health education rather than as population-level prevalence estimates or demonstrated causal benefits, and confirmation in larger, methodologically robust studies is warranted.

## Appendices

Final study questionnaire

The item list and response options below reflect the questionnaire exactly as electronically administered to participants via Google Forms and correspond precisely to the items and response categories analysed in Tables 3-5 of the Results section. (An earlier draft of this appendix, circulated internally during protocol development, contained additional items, e.g., separate female-susceptibility and adult-susceptibility yes/no items, and "rapid weight loss" and "pregnancy/hormonal factors" items, that were not included in the final administered instrument and were therefore removed from this appendix to keep it consistent with the reported results.)

Introduction

We are a group of medical students from Ibn Sina National College for Medical Studies. This study aims to assess awareness among adults in Jeddah regarding the link between obesity and gallstone formation. Your participation will help us understand the role of health education in preventing this condition. Participation is completely voluntary, and all your answers will be kept confidential and used for research purposes only. We appreciate you taking the time to complete this survey accurately.

Eligibility: Aged ≥18 years and currently residing in Jeddah.

Consent

Do you consent to participate? ☐ Yes  ☐ No (selecting "No" ends the survey)

A) Eligibility Check

1. Are you 18 years or older? ☐ Yes  ☐ No ("No" ends the survey)

2. Do you currently live in Jeddah? ☐ Yes  ☐ No ("No" ends the survey)

B) Participant Information

1. Age: ☐ 18-25  ☐ 26-35  ☐ 36-45  ☐ 45+

2. Gender: ☐ Male  ☐ Female

3. Nationality: ☐ Saudi  ☐ Non-Saudi

4. Educational background: ☐ High school  ☐ University  ☐ Postgraduate  ☐ Other

5. Employment: ☐ Employed  ☐ Student  ☐ Unemployed  ☐ Homemaker  ☐ Retired

6. Marital status: ☐ Single  ☐ Married  ☐ Other

7. Height (cm): ______   8. Weight (kg): ______  (used for BMI calculation only; not shown to participants)

C) Medical Background (Self-report)

1. Have you ever been diagnosed with gallstones? ☐ Yes  ☐ No  ☐ Not sure

   1a. If Yes - Treatment received: ☐ Surgery  ☐ Medications  ☐ Watchful waiting  ☐ Other

2. Do you have a close family member with gallstones? ☐ Yes  ☐ No  ☐ I don't know

3. Do you have diabetes mellitus? ☐ Yes  ☐ No  ☐ Not sure

4. Have you ever been told you have high cholesterol or triglycerides? ☐ Yes  ☐ No  ☐ Not sure

5. Physical activity in a typical week: ☐ 1-2 days  ☐ 3-5 days  ☐ 6-7 days

D) Knowledge and Awareness About Gallstones

1. Have you ever heard of gallstones? ☐ Yes  ☐ No (if "No", the survey ends here)

2. Do genetics play a role in gallstones? ☐ Yes  ☐ No  ☐ I don't know

3. Which gender is more susceptible to gallstones? ☐ Male  ☐ Female  ☐ Both equally  ☐ I don't know

4. Which age group is more susceptible to gallstones? ☐ Adults  ☐ Elderly  ☐ Children  ☐ I don't know

5. Does obesity play a role in gallstones? ☐ Yes  ☐ No  ☐ I don't know

6. What is the most common symptom of gallstones? ☐ Right upper abdominal pain  ☐ Nausea/vomiting  ☐ Yellowish skin or eyes  ☐ I don't know

7. Can a healthy lifestyle and exercise decrease the risk of gallstones? ☐ Yes  ☐ No  ☐ I don't know

8. Which type of food increases the risk of gallstones? ☐ Fatty food  ☐ Vegetables  ☐ I don't know

9. What is the main investigation used to diagnose gallstones? ☐ Ultrasound  ☐ X-ray  ☐ CT scan  ☐ I don't know

10. What is the usual treatment for gallstones? ☐ Surgery  ☐ Medications  ☐ Herbal remedies  ☐ I don't know

11. Can gallstones be asymptomatic? ☐ Yes  ☐ No  ☐ I don't know

12. What is the main complication of gallstones? ☐ Gallbladder inflammation  ☐ Kidney stones  ☐ Pancreatitis  ☐ I don't know

E) Risk Factors Perception

1. Do you have a family history of gallstones (in direct relatives)? ☐ Yes  ☐ No  ☐ I don't know

2. Do you think age is a risk factor for gallstones? ☐ Yes  ☐ No  ☐ I don't know

3. Do you think obesity increases the risk of gallstones? ☐ Yes  ☐ No  ☐ I don't know

4. Do you think high cholesterol is a risk factor for gallstones? ☐ Yes  ☐ No  ☐ I don't know

5. Do you think high triglycerides increase the risk of gallstones? ☐ Yes  ☐ No  ☐ I don't know

6. Do you think diabetes mellitus is a risk factor for gallstones? ☐ Yes  ☐ No  ☐ I don't know

7. Do you think low HDL ("good cholesterol") is a risk factor for gallstones? ☐ Yes  ☐ No  ☐ I don't know
